# The interactions between COVID-19 drugs and psychotropic agents

**DOI:** 10.1192/j.eurpsy.2021.63

**Published:** 2021-08-13

**Authors:** C. Hiemke

**Affiliations:** Department Of Psychiatry And Psychotherapy, Johannes Gutenberg-Universität Mainz, Mainz, Germany

**Keywords:** COVID-19, drug-drug interactions, pharmacokinetic, pharmacodynamic

## Abstract

Coronavirus disease (COVID-19) is a systemic infection targeting multiple organs. Interstitial pneumonia is the landmark feature of this condition. Severe acute respiratory symptoms requiring intensive care support arises for about one out of twenty symptomatic cases. Aminochinolone, antiviral, antibiotic, corticoid, anticoagulant and immunobiological drugs are used, mostly to treat symptoms. Only remdesivir exhibiting weak antiviral activity is approved for COVID-19. Psychotropic medications may interact with medical treatments for COVID-19. The aim of this presentation is to highlight pharmacokinetic and pharmacodynamic drug-drug interactions to be expected for medical treatments of COVID-19. Remdesivir and favipiravir exhibit hepatotoxic properties which may be enhanced under combinations with tricyclic antidepressants or agomelatine. Favipiravir, hydroxychloroquine, chloroquine, azithromycin, lopinavir/ritonavir have QT interval prolongation potential and must be considered for combinations with antidepressant and antipsychotic drugs. For hydroxychloroquine, hypoglycemic activity may give rise to endocrine disturbances. Pharmacokinetic drug-drug interactions can be expected for lopinavir/ritonavir which inhibit cytochrome P-450 (CYP) 3A4 and induce CYP2C9 and CYP2C19. Combinations with psychotropic drugs that are substrates of these enzymes (victim drugs) will affect drug concentrations in blood and lead to supra- or subtherapeutic levels. Moreover, it must be assumed that the COVID-19 infection is associated with an enhanced production of cytokines which has a known impact on CYP enzyme activities. Though studies on interactions between psychotropic medications and medical treatments for COVID-19 are lacking, multiple drug interactions can be predicted and expected considering the side effect profiles and CYP inhibitory, inducing and substrate properties of combined drugs.

**Disclosure:**

No significant relationships.

